# Toxigenic Fungi and Mycotoxins in a Climate Change Scenario: Ecology, Genomics, Distribution, Prediction and Prevention of the Risk

**DOI:** 10.3390/microorganisms8101496

**Published:** 2020-09-29

**Authors:** Giancarlo Perrone, Massimo Ferrara, Angel Medina, Michelangelo Pascale, Naresh Magan

**Affiliations:** 1 Institute of Sciences of Food Production (ISPA), National Research Council (CNR), 70126 Bari, Italy; massimo.ferrara@ispa.cnr.it (M.F.); michelangelo.pascale@ispa.cnr.it (M.P.); 2 Applied Mycology Group, Environment and AgriFood Theme, Cranfield University, Cranfield MK43 0AL, UK; a.medinavaya@cranfield.ac.uk

**Keywords:** climate change, mycotoxin, toxigenic fungi, biodiversity, provisional model, biological control, resilience, acclimatization

## Abstract

Toxigenic fungi and mycotoxins are very common in food crops, with noticeable differences in their host specificity in terms of pathogenicity and toxin contamination. In addition, such crops may be infected with mixtures of mycotoxigenic fungi, resulting in multi-mycotoxin contamination. Climate represents the key factor in driving the fungal community structure and mycotoxin contamination levels pre- and post-harvest. Thus, there is significant interest in understanding the impact of interacting climate change-related abiotic factors (especially increased temperature, elevated CO_2_ and extremes in water availability) on the relative risks of mycotoxin contamination and impacts on food safety and security. We have thus examined the available information from the last decade on relative risks of mycotoxin contamination under future climate change scenarios and identified the gaps in knowledge. This has included the available scientific information on the ecology, genomics, distribution of toxigenic fungi and intervention strategies for mycotoxin control worldwide. In addition, some suggestions for prediction and prevention of mycotoxin risks are summarized together with future perspectives and research needs for a better understanding of the impacts of climate change scenarios.

## 1. Introduction

Toxigenic fungi and mycotoxin occurrence varies between crops, as fungal species and strains differ in their ability to infect a particular plant host. Crop varieties also show different levels of susceptibility or resistance to toxigenic fungal infection. In addition, the same plant host can be attacked by different toxigenic fungi resulting in multi-mycotoxin contamination. Additionally, interacting climate-related abiotic conditions, especially water availability, temperature and perhaps atmospheric CO_2_, influence the infection of food crops by mycotoxigenic fungi and influence mycotoxin contamination levels.

In this respect, the last report of the Intergovernmental Panel on Climate Change (CC) revealed that warming of the climate system is unequivocal, with many of the observed changes over the last few decades being unprecedented [1]. Such climate changes could have significant impacts on the life cycles of toxigenic fungi and modify host–resistance and host–pathogen interactions. This could have significant impacts on the resilience of different toxigenic species and their ability to produce mycotoxins [2].

Climate represents the key agro-ecosystem driving force of fungal colonization and mycotoxin production [3]. Thus, changes in temperature, precipitation, and atmospheric CO_2_ concentration could lead to an unexpected increase/reduction of relative risk of mycotoxin contamination of crops in the field and post-harvest, which might have an impact on the geographical distribution of cereals and other staple crops, toxigenic fungi and their mycotoxins [4,5]. Indeed, global warming will not only have an impact on host–pathogen interactions already present in certain regions, but will perhaps favour the emergence of new diseases, and changes in the fungal biodiversity/microbiome because of fluctuations in their ecological niches. This will result in modifications of the distributional range, temporal activity and community structure of fungal pathogens [6,7]. In addition, the phenology and conditions of the hosts will be altered [8]. There is also evidence that fungal plant pathogens, including toxigenic species, are predicted to move globally and to change the diversity of diseases and pests invading staple crops with both economic and social implications and costs [9,10].

Climate change scenarios also act in slowly shaping the relationship between plant growth and associated fungal diseases and the traditional and classic balanced triangle between the pathogen/pest, host and environment is changing and becoming skewed [11,12]. In the context of toxigenic fungal pathogens, this could result in a switch from the so-called mycotoxin suppressive to conducive conditions or vice versa. One scenario could be of certain toxigenic fungi disappearing from the environment, while appearing in new regions previously never considered to be at risk. For example, this was clearly demonstrated by the increased risk of aflatoxins in Northern Italy and Eastern Europe in the last fifteen years [13]. Therefore, it is evident that both biodiversity and ecology of toxigenic fungi could be profoundly influenced by CC. Recent studies of the soil microbial biodiversity of grasslands showed how such CC-related impacts might occur [14]. In addition, fungal biodiversity is one of the most important traits that influence the occurrence and severity of mycotoxin contamination, because of the phenotypic and metabolic plasticity of toxigenic fungi. New and emerging combinations of mycotoxins in food/feed represent the ability of fungi to adapt to such changing conditions [15,16]. In this respect, the effect of three-way interactions amongst environmental factors (temperature × water availability × CO_2_) on the ecophysiology of toxigenic fungi and mycotoxin accumulation has been recently investigated by several authors [10,17,18,19,20,21]. Nevertheless, obtaining a clear picture of the influence of climate on overall crop contamination by mycotoxins and on toxigenic fungal ecology and adaptation is complex and frequently intractable; so the real effects of CC are difficult to ascertain. It is also problematic to distinguish the long-term effects of climate on toxigenic fungal evolution in agricultural crops from short-term effects of seasonal changes and agronomic practices. This will be possible when longer-term sets of data are available on the biology and ecology of toxigenic fungi in association with CC and extreme weather events. In this respect, a comprehensive and interesting review on articles published during the past 30 years relating to the potential climate change effects on plant pathogens and crop disease risks has recently been published [22]. However, the available documented information during the last decade has resulted in the development of a new area of investigation; specifically, examining the impact of CC-related scenarios on the resilience of key mycotoxigenic fungi and their ability to produce mycotoxins in staple food chains. In this regard, predictive models using historical or current climatic condition datasets have been performed in order to estimate, in a more realistic way, the impact of CC factors on fungal colonization and mycotoxin contamination [23,24,25]. These studies applied models of different disciplines including climate projection, crop phenology and fungal/mycotoxin prediction in crops, but the limited number of models taking into account all of the CC-related abiotic factors, and their limited validation in only certain regions globally suggests that improvement of such predictive approaches is needed. Thus, more research is required on the impact of the three-way CC-related abiotic factors and the host plants on mycotoxin accumulation [26]. In addition, we may be heading for a ‘‘new age of extinction”, which will surely apply to fungi, including mycotoxigenic moulds [27]. Could some mycotoxigenic fungi become extinct? There is a need not only for interdisciplinary collaboration between phytopathologist, mycologist, agronomists and climate scientists, but also for more awareness of investigations relating to CC, especially interactions between increased temperature and exposure to elevated CO_2_ and wet/drought stress and the resilience of mycotoxigenic fungi, including how rapidly they are able to become acclimatized and whether this influences the predominant mycotoxin production patterns, or whether this results in new secondary metabolite production patterns. However, there is still a significant number of unanswered questions that need to be addressed for a more comprehensive and informed view of how CC impacts key mycotoxigenic fungi and their contamination of staple food/feed crops with mycotoxins. For example: are current control/mitigation/intervention strategies going to be effective in the future? What impact will CC-related abiotic stresses have on interactions and relative dominance of toxigenic fungi and other phyllosphere or rhizosphere microbiota? Will existing agricultural practices, including Good Agricultural Practices (GAP) and Hazard Analysis Critical Control Point (HACCP), have to be changed in order to minimize the mycotoxin contamination when environmental shifts and CC-related fluxes become the norm? [12,28]. More research is urgently required to address these key questions to effectively predict the level of risk of different mycotoxins in economically important staple food crops in the future and to understand whether they are resilient enough to tolerate the expected CC-related conditions [10].

The authors of the present paper wish to give an opinion-piece by summarizing a general overview of the main results obtained in the last decade on ecology, genomics, distribution of toxigenic fungi and mycotoxins in CC-related scenarios with suggestions for prediction and prevention of the above highlighted risks. In addition, the authors wish to underline the future perspectives and research needs for a better understanding of the impacts of these scenarios.

## 2. Biodiversity, Ecology and Resilience of Toxigenic Fungi in Climate Change Scenarios

As briefly discussed in the introduction, it is evident that both biodiversity and ecology of toxigenic fungi could be profoundly influenced by interacting CC abiotic factors; in fact, determining the temporal scaling of biodiversity in the face of global climate change is a central issue in ecology; this was recently studied for the soil microbial biodiversity of grassland [14]. They examined the species–time relationships and phylogenetic–time relationships of soil bacteria and fungi in a long-term multifactorial global change experiment with warming (+3 °C), half precipitation (−50%), and double precipitation (+100%). The main evidence was that bacteria and fungi had higher overall temporal scaling rates with respect to plants and animals, showing the importance of monitoring these aspects in a warming world. In addition, fungal biodiversity was one of the most important traits that influences the occurrence and severity of mycotoxin contamination, due to phenotypic and metabolic plasticity that it confers to toxigenic fungi.

New and emerging combinations of mycotoxins in food/feed represent the ability of fungi to adapt to the changing conditions [15,16]. Therefore, it is very important to know the biodiversity of species that can contaminate a crop and the change in composition that can occur in a CC scenario. In general, based on present available data, atmospheric concentrations of CO_2_ are expected to double or triple (from 350–400 to 800–1200 ppb) in the next 25–50 years. Thus, different regions worldwide will be impacted by the increases in temperature of 2–5 °C, coupled with elevated CO_2_ (800 to 1200 ppm) and more frequent drought episodes, which in turn might have a profound effect on mycotoxigenic fungi and mycotoxin production [9].

Recent studies have examined the changes in fungi biodiversity by developing new methodologies for assessing toxigenic–pathogenic and beneficial fungi in a specific region or ecosystem, leading to a quantitative risk–benefit assessment strategy in relation to CC conditions [29]. The impact on microfungal biodiversity of CC was studied in Slovakia vineyard regions, demonstrating important effects of the climatic conditions on microfungal biodiversity [30]. In addition, it has been predicted that in Europe in the next 50–100 years, *Aspergillus flavus* may outcompete *A. carbonarius*, with aflatoxins (AFs) becoming a greater risk than ochratoxin A (OTA); similarly, the conditions may become too hot for *Penicillium expansum* and reduce the threat from patulin in pome fruits [5]. However, whether and how CC affects microbial species–time relationships remains unclear, mainly due to the scarcity of long-term experimental data that needs to be implemented for a better understanding of changes in microbial biodiversity. Climate change can influence variability at different stages of the fungal life-cycle, including sporulation, and lead to modifications in fungal diversity and the dynamics of their performance. There are indications that changing climate may lead to alterations in phenology and dynamics of fungal communities, but the impact of global change on fungal ecology is still not well understood [31].

Biodiversity is strictly related to the ecology of the fungi and the ecological niches they occupy. In this regard, the life cycles of key mycotoxigenic fungi are relatively well understood, including growth phases, pathogenicity and saprophytic components and effects of abiotic conditions on toxin contamination on different staple crops, especially under existing abiotic conditions. However, less is known about how interacting conditions of temperature stress (+2–5 °C), exposure to elevated CO_2_ (400 vs. 1000–1200 ppb) and water availability extremes may affect colonization and toxin production. For example, growth patterns of *Fusarium* species such as *F. graminearum* and *F. verticillioides* were shown to change under such interacting CC-related conditions in vitro [10]. In situ infection of ripening maize by *F. verticillioides* was shown to increase when exposed to elevated CO_2_ and temperature during silking of maize plants based on biomass quantification. However, fumonisin B_1_ (FB_1_) contamination was not shown to increase [17]. Subsequent studies including drought stress showed that this influenced FB_1_ production and actually stimulated contamination of ripening maize cobs, partially due to physiological effects on the maize plant [32]. Studies of ochratoxigenic species such as *A. carbonarius*, *A. westerdijkiae*, *A. steynii* and *P. verrucosum* on different matrices have also examined effects of CC-related abiotic factors on growth and toxin contamination [18,21,33,34]. Overall, the growth rates of the *Aspergillus* section *Circumdati* and *Nigri* species was not affected by CC-related interacting abiotic factors. However, in vitro experiments, both on coffee-based media and on stored coffee beans, suggested that there was a stimulation of OTA production (e.g., *A. westerdijkiae*), while for other species (e.g., *A. carbonarius*) there was a reduction in toxin contamination [18]. Studies by Cervini et al. with strains of *A. carbonarius* isolated from grapes showed that while growth was relatively unaffected on grape-based media by CC-related interacting factors (+4 °C, 400 vs. 1000 ppm CO_2_, water stress), toxin production was stimulated for some strains, and this was confirmed with an increase in relative gene expression of cluster genes involved in toxin biosynthesis [21].

A significant amount of attention has been given to *A. flavus* and the impacts that aflatoxin contamination of maize under existing and expected CC-related abiotic factors. Recent studies of colonization and aflatoxin B_1_ (AFB_1_) contamination of maize cobs of different ripening stages (representing different water availabilities) showed that both lesion size and toxin contamination were similar at 37 °C when compared with the supposed optimum conditions of 30 °C [35]. This suggests that when maize is superimposed with elevated CO_2_ concentrations, additional changes in toxin contamination may occur. Studies in vitro and on harvested maize grain have shown that growth is relatively unaffected, while AFB_1_ is significantly stimulated when exposed to CC-related abiotic factors compared to existing conditions (30 vs. 37 °C; 350–400 vs. 1000 ppm CO_2_; 0.995 vs. 0.93 water activity). Indeed, this was supported by the increased relative expression of biosynthetic genes involved in toxin production [28,36]. Further transcriptomic data analyses showed changes in secondary metabolite gene clusters (aflatoxins, cyclopiazonic acid), universal regulators, sugar transporters and other stress-related pathways. This suggests that there may be switches in secondary metabolite production patterns under CC-related conditions [28,37]. In addition, the effect of increases in temperature and CO_2_ concentration was recently studied in *Alternaria alternata* strains by Siciliano et al. [38]. They used four different strains, three different host plants, and six different combinations of increased T range (day/night) and CO_2_. In general, the increase of temperature significantly influenced the disease index, while mycotoxin production was very variable and influenced by strain and host plant factors. In particular, tenuazonic acid was the most frequent mycotoxin produced amongst the four toxins quantified.

Overall, the available information suggests that there may be differential effects of CC-related interacting abiotic factors on individual mycotoxigenic species and that in depth knowledge of potential impacts are needed on strains of a specific species before making predictions of the impact of CC factors in relation to an increase or decrease in mycotoxin production. It is possible that if the temperature increases and water supplies decrease in the future, the adaptation of fungi by mutation and sexual recombination would give some advantages for survival of resilient species. Warmer climates favor thermotolerant species, and this will lead to dominance by toxigenic *Aspergillus* species over *Penicillium* species, as well as perhaps changing the relative production of mycotoxins by the same species, for example, *A. flavus* switching production of aflatoxins to cyclopiazonic acid. Thus, fungal community structure and diversity may change significantly influencing the relative mycotoxin contamination in the food chain. However, there are few studies on the impact that acclimatization of crops, or fungal pathogens and pests, may have on the plant/disease pest interface. Some preliminary work by Medina et al. on the influence of acclimatization on *A. flavus* strains in comparison with non-acclimatized strains in terms of growth and AFB_1_ production showed that for one strain, growth and AFB_1_ production was significantly stimulated [28]. This suggests that there may be intra-strain differences in the effects of acclimatization, where mycotoxin production could be extremely variable on the basis of a mixed fungal population that has differently acclimatized. Additional studies on toxigenic fungi acclimatization and interactions with other mycobiota are necessary to obtain more accurate data on implications for mycotoxin contamination on food under CC scenarios.

## 3. Molecular Characterization and Genomic Evidence on Toxigenic Fungi in a Climate Change Scenario

The study of molecular ecology of toxigenic fungi will be beneficial in understanding the effect of CC on toxigenic risk and will point out the resilience of such fungal pathogens under expected CC conditions. Recently, there has been interest in the resilience of mycotoxigenic fungi, including several *Fusarium* and *Aspergillus* species.

Different molecular approaches have been used to elucidate the relationship between these interacting environmental factors and mycotoxin production. The availability of modern, high-throughput techniques, including genomics, transcriptomics, metagenomics, proteomics and metabolomics has contributed to a rapid expansion of data on the biology of mycotoxigenic fungi. This has facilitated a significant increase in our knowledge of the biological and biochemical processes regulating the production of mycotoxins, and the adaptation of these fungi to environmental stresses.

Several studies have examined the impact that environmental factors have on the fumonisin (FUM) cluster genes and the effects on FB_1_ and FB_2_ production by a different Fusaria. In *F. verticillioides*, it has been shown that temperature and water activity (a_w_) have a significant effect on gene expression of key biosynthetic genes, growth, and the phenotypic production of toxic secondary metabolites [39]. Studies on the ecology of *F. langsethiae* have compared different strains from northern European countries and identified optimum conditions for growth as being between 0.98 and 0.995 a_w_ and 25 °C [40], while production of T-2+HT-2 was highest at 20–25 °C and 0.995 a_w_ [41]. Studies on stored oats colonized by *F. langsethiae* showed different results when exposed to CC abiotic conditions, especially when grown at 30 °C, intermediate water stress (0.98 a_w_) and 1000 ppm CO_2_ exposure. Under these conditions, there was a stimulation of *Tri5*, *Tri6* and *Tri16* gene expression and a significant increase in T-2/HT-2 toxin contamination when compared to the controls (20 °C, 0.995 a_w_, 400 ppm CO_2_) [19]. Indeed, there was a correlation between the relative expression of these genes and mycotoxin production. However, *F. langsethiae* appears to be less resilient than other Fusaria such as *F. graminearum* and *F. verticillioides* based on data in relation to temperature with a_w_ stress, and in some cases, interactions with CO_2_ exposure [39,42,43].

In *F. culmorum* and *F. graminearum*, previous studies using a mycotoxin microarray with sub-arrays for specific mycotoxins have demonstrated that for relative trichothecene (TRI) gene expression under different environmental conditions, growth and deoxynivalenol (DON) production are strictly correlated and it is possible to integrate such microarray data with ecological data to develop models that can be used to predict DON concentrations [44].

Studies with *A. flavus* colonizing maize grain showed that under expected CC conditions, while growth was relatively unaffected, structural and regulatory biosynthetic genes involved in mycotoxin production (such as *aflD* and *aflR*) and phenotypic aflatoxin B_1_ production were stimulated under the three-way interacting CC conditions represented by temperature × a_w_ × CO_2_ [28,36,37]. These findings were in agreement with previous evidence demonstrating that the expression of key genes in the biosynthesis of aflatoxin, including the key regulatory genes (*aflR*, *aflS*), enabled a relatively accurate prediction of aflatoxin production at increased temperatures and water stress conditions relevant to climate change scenarios [45].

Zhang et al. examined *A. flavus* activity and the transcriptome at 0.99 and 0.93 a_w_ on a conducive Yeast Extract Sucrose (YES) medium at 28 °C. They found both decreased conidiation and AFB_1_ biosynthesis at 0.93 a_w_ when compared to 0.99 a_w_. Overall, their study showed 5362 differentially expressed genes identified between 0.99 and 0.93 a_w_ treatments, including 3156 up-regulated and 2206 down-regulated genes. They suggested that *A. flavus* underwent an extensive transcriptome response during a_w_ variation between freely available water and water stress [46].

Yu et al. used the RNA-Seq approach to reveal transcriptome changes and aflatoxin regulation in *A. flavus* grown at 30 and 37 °C. They found 1153 genes that were differentially expressed at 30 and 37 °C. Eleven of the 55 secondary metabolite clusters were up-regulated at the lower temperature, including aflatoxin biosynthesis genes, which were among the most highly up-regulated genes. On average, transcript abundance for the 30 aflatoxin biosynthesis genes was 3300 times greater at 30 °C as compared with 37 °C. These results supported the hypothesis that high temperature negatively affects aflatoxin production by turning down transcription of the two key transcriptional regulators, *aflR* and *aflS* [47]. However, these were very short-term studies using defined liquid broth-based media.

More recently, Medina et al. investigated the effect of interacting a_w_ × temperature (0.99/0.91 a_w_ and 30/37 °C) on *A. flavus* colonizing stored maize grain using transcriptomic analyses, which showed some correlation with AFB_1_ production [28]. There were higher expression levels of most aflatoxin biosynthetic genes under extreme environmental stress conditions in terms of increased temperature and lowered a_w_, as well as an increase in AFB_1_ levels. Similar effects were observed on transcript levels when only looking at changes in temperature or a_w_ levels alone. Furthermore, the analysis of *aflS*, *aflR*, *aflB*, and *aflT* showed an up-regulation in their response to water stress (0.91 a_w_) at 37 °C compared to 30 °C, indicating key genes in the aflatoxin cluster may be more sensitive to water stress at higher temperatures. Additionally, among the five most highly up-regulated genes, the *aflF*, *aflU* and *aflT* genes are adjacent and located on the very end of the gene cluster, whereas *aflG* and *aflNa* are located next to each other in the middle of the gene cluster. Therefore, the gene *aflF* could be related to switching the aflatoxin pathway gene expression on/off, and, based on chromosomal location, these genes may be responsive to the environmental queue of water stress.

In *A. carbonarius*, several ecophysiological studies simulating field conditions revealed that fungal growth and OTA production were determined by environmental factors such as temperature, water availability and photoperiod [21,48,49]. Additionally, in a recent study by Cervini et al., the effect of two different day/night temperature cycles (15–28 °C vs. 18–34 °C, 11.5 h/12.5 h dark/light), and two CO_2_ concentrations (400 vs. 1000 ppm) were evaluated [20]. The authors revealed that the combination of the 15–28 °C temperature cycle with elevated CO_2_ level (1000 ppm) resulted in an overall up-regulation of biosynthetic genes (*AcOTApks*, *AcOTAnrps*, *AcOTAhal*, and especially *AcOTAbZIP*, encoding for a transcription factor), which were positively correlated with the phenotypic production of OTA under this growth condition. Moreover, *laeA*, *veA*, and *velB* genes were up-regulated following a similar expression pattern of the other structural genes, suggesting that the 15–28 °C × 1000 ppm CO_2_ condition may directly influence the global regulatory velvet complex and thus OTA production. Regarding the *Aspergillus* section *Circumdati* species and *Alternaria* genus, although several studies have investigated the effects of different temperatures × CO_2_ levels on *A. westerdijkiae*, *A. niger*, *A. ochraceus*, *A. steynii* and *Alternaria* species/strains [18,33,38], to date, no data are available on toxin regulations at a molecular level, and this gap in knowledge needs to be addressed.

Under expected CC conditions, another key aspect that should be investigated in more depth is the effect of such interacting abiotic stresses at an epigenetic level. Recent studies indicate that many fungal secondary metabolites are regulated by epigenetic modifications, which not only affect the titers of secondary metabolites, but also activate cryptic gene clusters [50]. With genomes being available, epigenomic studies have been increasing in the last few years. Regarding toxigenic fungi, preliminary data on the regulation of mycotoxin production at an epigenetic level are already available. In *A. flavus*, methylation regulators such as *RmtA* and *DmtA* are described as epigenetic factors involved in fungal development and mycotoxin production [51,52]. Another example is the impact of chromatin remodeling on *fum1*, *fum21* and *fum8* expression in fumonisin production by *F. verticillioides* [53]. Indeed, a better understanding of how chromatin remodeling is influenced under interacting environmental factors, and the integration of these findings with transcriptome analysis, ecology and mycotoxin production, will contribute to a more comprehensive view of the molecular aspects involved, not only in mycotoxin production, but also in resilience to CC. Toxigenic fungal resilience is strictly linked to the possible impact of acclimatization of toxigenic fungi to CC conditions. In addition, this aspect needs to be examined in terms of whether this will influence colonization rates of important commodities by toxigenic fungi and whether this may lead to enhanced or reduced mycotoxin contamination.

## 4. Distribution of Mycotoxins Worldwide—New Risk Maps Due to Climate Change

Mycotoxin contamination of food and feed, from raw materials to processed foods, is continuously reported worldwide. Aflatoxins (AFs), deoxynivalenol (DON), ochratoxin A (OTA), fumonisins (FBs), T-2 and HT-2 toxins (T-2, HT-2), patulin (PAT) and zearalenone (ZEA) are considered to be significant from a food/feed safety point of view, with aflatoxins the most important mycotoxins due to their genotoxic and carcinogenic effects, with the most contaminated commodities being cereals (mainly maize and wheat) and different types of nuts.

The majority of mycotoxins of concern to human and animal health are associated with filamentous fungi of the genera *Aspergillus*, *Fusarium* and *Penicillium*. These fungi under favourable environmental conditions, such as temperature and humidity, are able to produce toxic metabolites in the field, as well as in storage conditions. Since their discovery, *Aspergillus* spp. (AFs and OTA producers) have been shown to have a worldwide distribution but primarily occupy tropical/sub-tropical regions growing at high temperatures and lowered water activity. *Penicillium* spp. (OTA and PAT producers) grow and produce mycotoxins over a wide range of temperatures, more abundant in temperate climatic regions and are commonly associated with storage. *Fusarium* spp. (DON, FBs, T2, HT-2 and ZEA producers) adapt to a wide range of habitats and have a worldwide distribution; they are important plant pathogens and a few species are significant mycotoxin producers (see Table 1). Consequently, the occurrence of different mycotoxins in the field is related to the geographical location where the crop is grown and to meteorological conditions, although Good Agricultural Practices (GAPs) and Good Manufacturing Practices (GMPs) during the handling, storage, processing, transportation and distribution play an important role in minimizing mycotoxin contamination.

Over the years, several review papers and reports have been published that estimate the risk associated with mycotoxin contamination in agricultural crops, processed food and feed products. Occurrence data from international organizations such as the Food and Agriculture Organization (FAO), European Food Safety Authority (EFSA) or World Health Organization (WHO) databases are mainly related to processed food and feed samples and have contributed to risk assessment studies aimed at evaluating risks of human and animal exposure to mycotoxins. It should be remembered that food and feed companies produce “safe” commodities that must comply with established regulatory limits. Data relevant to field contamination (analysis of raw materials) are necessary to evaluate the year-to-year risk from mycotoxins or to evaluate the effect of climate change on mycotoxin prevalence. These data come mainly from research papers, while data from private companies are not published or submitted to WHO, EFSA or FAO.

Previously reported data on mycotoxin occurrence aimed at the evaluation of the intake of mycotoxins and the risk associated with their ingestion by populations (i.e., JECFA, WHO and SCOOP reports) have shown a widespread occurrence of mycotoxins around the world with a high variability in frequency and levels of contamination, depending on the commodity, the cropping season and the geographical region. Recently, an extensive data collection from 104 papers published over 10 years since 2006 showed that mycotoxins are ubiquitously present in cereals and cereal-based products [54]. Aflatoxins continue to be a significant problem in Africa (incidence rate of 50%) and Asia (63%). A low rate of contamination was observed in South America (15%), while in Europe the incidence of AFs was increasing (44% of samples contaminated), with maize the most contaminated cereal. Lower levels of contamination were found in processed products, compared to raw materials, indicating that the risk for consumers is low. Deoxynivalenol is a mycotoxin commonly found in wheat in all countries, with contamination rates from 50% in Asia to 76% in Africa. Similar extensive DON contamination in cereals has been reported worldwide in past surveys. Like DON, FBs are largely diffuse over the world in maize and maize-based products. An incidence of 62% was reported in samples from Africa and Asia. The highest incidence was observed in North and South America (up to 95%), while Europe had the lowest incidence of FBs (39%), although levels of contamination higher than the legal limits were found. A large variation of incidence of ZEA worldwide has been reported. This showed a low incidence of ZEA in Asia (15%) and the highest in Africa (59%). The incidence of positive OTA samples was variable, with a range from 20% in Europe to 42% in North America. The most contaminated products are oats and oat-based breakfast cereals [54].

In 2004, Biomin Inc. (Austria) started a global survey program to monitor mycotoxin contamination of animal feeds. Today, this database is a valuable source that is able to provide a comprehensive overview on the worldwide occurrence of mycotoxins in raw materials, i.e., agricultural commodities used for livestock feed. These data are available online (https://www.biomin.net/solutions/mycotoxin-survey/) or have been published in several peer-reviewed journals. A recent paper published by Biomin analyzed a large-scale 10-year survey of mycotoxin contamination in feed at a global level and assessed regional differences in the year-to-year variation of mycotoxin occurrence [55]. A total of 74,851 samples of feed and raw material intended for feed purposes were collected from 100 countries and analyzed for the major mycotoxins over the period 2008 to 2017. A high frequency of mycotoxin contamination (88%) was found with a large fraction of samples (64%) co-contaminated with at least two mycotoxins. The *Fusarium* mycotoxins DON, FBs and ZEA were most prevalent, compared to AFB_1_, OTA and T-2 toxin. Data were analyzed at a regional level by sub-dividing the globe into 15 geographical regions. DON was the most prevalent mycotoxin in several regions, mainly in Europe (Northern 74%, Central 70%, Southern 53% and Eastern 60%), North and Central America (64% and 70%, respectively), South Africa (63%) and East Asia (85%). FBs were found in all regions with Southern Europe, Central and South America, Middle East and North Africa, Sub-Saharan Africa, South Africa, South Asia and East Asia, exceeding 60% of contaminated samples. ZEA was found mainly in Central Europe (45%), Eastern Europe (42.5%), South America (47%), Middle East/North Africa (45%), Sub-Saharan Africa (52%), South Africa (72%), Southeast Asia (46%) and East Asia (61%). OTA was sporadically present, mainly in Southern and Eastern Europe (21% and 36%, respectively) and Sub-Saharan Africa (32%). The highest incidence of positive samples was from South Asia (60%). Concerning T-2 toxin, it was mainly present in samples from Europe (from 12% to 48%). Aflatoxin B_1_ was prevalent in samples from Sub-Saharan Africa (76%), Southeast Asia (57%) and South Asia (82%, which was the highest percentage of positive samples found in any region), and was also detected at high concentrations. Interestingly, incidence rates from 22 to 29% were observed in samples from Southern Europe, South America and Middle East/North Africa.

As climatic conditions (i.e., temperatures and extreme changes in rainfall/drought episodes) are the main determinants for mycotoxin accumulation in crops, year-to-year variation of mycotoxin incidence and levels of contamination was observed in maize grown in 2013–2017. Mycotoxin concentration varied from year-to-year in several regions and it was influenced by the variation in weather conditions. For example, in Southeast Asia, East Asia and Central Asia in 2017 high levels of AFB_1_ were observed compared to 2013–2016, due to higher rainfall and temperatures during the silking period of maize. Rainfall and mild temperatures during the flowering and maturation period were the cause of higher DON and ZEA levels in maize grown in 2014 in Central and Southern Europe, compared to 2013, 2015, 2016 and 2017. In contrast, the low AFB_1_ levels found in samples from East Asia reflect the low precipitation which occurred during the flowering period. In Central Europa FBs concentrations were high in 2015 due to warmer temperatures and lower rainfall during silking and prior to harvesting of maize than in previous years. Similarly, a peak in FB concentration was observed in East Asian maize harvested in 2017 due to high temperatures in July [55].

This paper showed regional trends of mycotoxin contamination that were affected by the weather conditions, in particular when these changes occurred during the critical ripening stage development periods of the crops. In the future, climate changes could certainly significantly influence the levels and incidence of individual and mixtures of mycotoxin contamination. Climate change has indeed been suggested worldwide as a driver of emerging risks for food and feed safety, as well as for plant, animal health and nutritional quality [16,42,56,57]. An increased risk of contamination by mycotoxins in Europe in maize, wheat and rice has been recently considered by EFSA and potentially linked with climate change [2,56]. Existing provisional models have shown that AFB_1_ is predicted to become a food safety issue in maize in Europe, and as a consequence, in milk due to aflatoxin M_1_ contamination [13,58,59]. As an example to support this, in recent years AFs have become a problem in countries where aflatoxin contamination was previously not considered a risk. High levels of AFs have been found in maize intended for feed grown in Italy and Serbia in 2003, 2012 and 2015 cropping seasons [60,61,62].

Previous reports of the Joint FAO/WHO Expert Committee on Food Additives [63,64,65] together with recent papers that analyzed data (500,000 analyses) from EFSA and large global surveys available in the literature and in international databases suggest mycotoxin prevalence of >25% as stated by the FAO in 1999 [66,67]. In addition, for some mycotoxins, this figure may greatly underestimate the occurrence above the detectable levels. The high occurrence could be due to improved detection limits of the analytical methods used for mycotoxin analysis and due to the impacts of climate change. However, most of the mycotoxin occurrence surveys are not harmonized in terms of sampling procedures or analytical methods used. Consequently, it is quite difficult to compare datasets to develop reliable risk maps, mainly in a changing climate scenario. In addition, the totality of mycotoxin occurrence data come from governmental or literature sources, while a large quantity of data is produced by food and feed companies that are not published or submitted for peer review by international organizations (i.e., WHO, FAO, EFSA). Furthermore, occurrence data from many developing countries are limited or absent. Another aspect to be considered is the localization of the infection by toxigenic fungi in the field. This means that an accurate sampling plan should be carried out to obtain representative samples for mycotoxin analysis. The outcomes of these considerations lead to a high uncertainty in defining maps of risk for mycotoxins at a national, regional and global level based on occurrence data.

## 5. Provisional Model and Historical Data in Relation to a New Possible Scenario of Climate Change and Mycotoxin Diffusion

Provisional models often focus on weather forecasts and the susceptibility of the planted cultivar. Modelling mycotoxin production is more difficult with respect to foreseeing disease incidence and severity since toxin production is affected by additional factors, e.g., strain pathogenicity, toxigenicity, competition with other microbes in the plant, and effects of fungicides on toxin biosynthesis [68,69]. Most of the existing models are empirical in nature, as the fundamental factors connecting disease progression and toxin production to the environment are not well understood. Existing forecasting systems are based primarily on weather data, e.g., temperature, rainfall and moisture, and have been developed for application in particular geographic regions, usually where they were developed. In addition, most of the papers studying models and mycotoxins investigate how ecological factors influence fungal growth in controlled ecological conditions (different T and a_w_ levels), on artificial defined or heterogeneous commodity-based media. The results obtained define and describe, with mathematical functions, the effect of each variable considered on the fungal life-cycle and mycotoxin production giving a significant contribution to the development of predictive modeling of mycotoxin risks in crops [24]. The model developed could be empirical, or mechanistic. Only field data are considered for empirical models, while in vitro data significantly contribute to mechanistic model development. Both empirical and mechanistic models are used to predict mycotoxin contamination in crops. The mechanistic ones can be used irrespective of the geographic area, and thus seem to be more appropriate for the development of a general Decision Support System [70]. However, the combined use of more than one model usually gives more precise predictions [24]. Regarding studies on mycotoxin modeling and climate change we are still in the infant stages of development. Some recent work has developed models to predict climate change scenarios and their impacts on risks of mycotoxins in cereals in Europe by an empirical approach for deoxynivalenol (DON) in wheat [25] and a mechanistic model to predict aflatoxins (AFs) in maize and in pistachio nuts [24,71].

In general, modelling of the impacts of CC-related individual and combined abiotic factors on potential mycotoxin contamination have focused on the use of multidisciplinary historic datasets based on climate predictions, crop phenology and fungal infection/mycotoxin production pre-harvest, especially for wheat and maize, in relation to DON and AFs, respectively [13,23,25,72]. Van der Flels-Klerx et al. also examined *Alternaria* and its mycotoxins in perishables such as tomatoes [26]. Unfortunately, many of these models have included variations in single CC-related abiotic factors or sometimes two, but not all three. Thus, temperature increases of +2 °C and +5 °C, or exposure to increased CO_2_ levels have received attention [13]. For DON in wheat predictions, two empirical models were integrated to include wheat phenology and toxin contamination in some regions of Europe. In addition, climate model projections for 2031–2050 were included from various available databases. This included projected temperature fluctuations and rainfall patterns, solar radiation and estimates based on 50 × 50 km grids across the areas of interest. Unfortunately, exposure to elevated CO_2_ changes were not included in these databases. These suggested that in CC-related abiotic conditions there would be a 1–2 week earlier development of flowering and maturation of winter wheat, with DON contamination potentially increasing in most of the regions of Europe based on the simulations [25,26].

Battilani et al. used the IPCC CC models based on an increase of +2 °C and +5 °C to examine impacts on AFB_1_ in maize wheat and rice in Europe [13,23]. In these studies, meteorological datasets were obtained from the LARS weather generator and again a 50 × 50 km grid system was used across Europe. They used a mechanistic aflatoxin (AFLA-maize) model based on predictions of *A. flavus* infection and AFB_1_ contamination on a daily basis [13]. This produced an Aflatoxin Risk Index (AFI), linked to AFB_1_ contamination observed in field measurements. The inputs into the model showed that the areas in which maize would be cultivated would be larger across Europe. The AFI showed that there would be significantly wider regions of Europe that had a potentially higher level of risk from AFB_1_ contamination. The hot spots for these risks included central Spain, Italy, Hungary, Slovakia, the Czech Republic and the Balkans for the +2 °C scenario, and a much wider range of at risk regions in the +5 °C scenario, although in the latter case the relative AFB_1_ contamination would be less.

Recently, a mechanistic model was used to examine *Alternaria* infection of tomatoes in two regions of Europe, in Spain (Extramadura region) and in Poland (Krobia), using the weather inputs under different CC scenarios [26,73]. These models showed that in Spain the temperature changes (18.2–38.2 °C) would minimize growth of *Alternaria* and mycotoxin contamination because at the higher temperatures, most species of this genus cannot grow or produce mycotoxins [74]. However, in Poland, under the projected temperatures (14.2–28.4 °C), there would be an increase in *Alternaria* contamination with the range being within the optimum for production of alternariol, its monomethyl esters, altenuene and tenuazonic acid [74].

Van Der Fels-Klerx et al. [26] suggested that there were specific gaps in relation to predictive modelling of CC-related abiotic impacts on mycotoxin contamination. For example, very little validation of these models, with the exception of the few studies mentioned previously, has been done. Perhaps more accurate inputs on the life cycle of these toxigenic species and the impacts of temperature × CO_2_ × water availability interactions, especially the boundary conditions for growth/toxin production, are necessary to improve the robustness of the empirical models. Thus, more detailed input information is required for the predictive models being developed. The potential for more accurate prediction really depends on the interface between the resilience of the food plant under future CC scenarios, and that of the toxigenic species. Furthermore, while for some toxigenic species, toxin production remains similar under the forecasted CC-related conditions, for others, there can be significant effects, e.g., increasing toxin production or a switch in the major mycotoxin produced or the ratio of different mycotoxins. More knowledge is thus required to obtain a better understanding of the fungal and plant ecophysiology and the pathogen/host interface to improve the potential for making more accurate and relevant global predictions on the impact of mycotoxins on the food security of staple food crops under the forecasted conditions. Thus, in general, better understanding and modelling of the impact of climate change requires additional experimental data. The model could need re-calibration in changing conditions, and probably, as we are heading toward a changing world, mechanistic models should be more suitable than empiric ones [13]. Vaughan et al. suggested that modeling approaches combining data on climate, pathogen and host, including cropping systems, could provide great support in the estimation of disease and mycotoxin risk in anticipated scenarios. However, it is timely for more efforts to model the predicted impacts of future climatic situations and challenges, especially on specific mycotoxin-crop combinations, which would be very beneficial in quantifying potential risks in each commodity [32,75].

## 6. Effect of Acclimatization to the Predicted Conditions

There have been very few studies to examine the impact that acclimatization of toxigenic and indeed other fungal plant pathogens may have on pathogenicity or on toxin contamination of staple food products. Studies by Vary et al. [76] showed that wheat exposed to existing and CC-related CO_2_ levels (390 vs. 780 ppm) affected both the physiology of wheat including leaf physiology and stomatal patterns. They examined the effect of acclimatization of *F. graminearum* for 10–20 generations in elevated CO_2_ concentrations. They found that there was an increase in pathogenicity and disease progression of the *Fusarium* head blight symptoms. This was supported by molecular pathogen biomass quantification. Unfortunately, this was not combined with quantification of DON and its associated toxins. This would have provided useful knowledge in relation to the impacts of acclimatization on potential increased or decreased toxin contamination. Inclusion of elevated temperature and drought stress would have also been interesting.

Recent studies on aflatoxigenic *A. flavus* strains isolated from pistachios were found to grow optimally at 35 °C and 0.98–0.95 a_w_ on a pistachio nut-based medium and on pistachio nuts [77]. Strains were acclimatized at 37 °C and 0.98 a_w_ for 5 generations at 1000 pm CO_2_ on a pistachio nut-based medium. These were then subsequently compared with the non-acclimatized strains in terms of growth and AFB_1_ production under control conditions (400 ppm CO_2_/35 °C/0.98 a_w_) and under CC-related abiotic conditions (1000 ppm/37 °C/0.98 a_w_) on layers of pistachio nuts. In these studies, one acclimatized strain colonized pistachio nuts more rapidly than under existing conditions and for the other strain there was no effect. However, for the former strain, AFB_1_ production was also significantly stimulated, especially after 10 days colonization of pistachio nuts [28]. For the other strain there was no effect on AFB_1_ production. This certainly suggests that there may be intra-strain differences in effects of acclimatization, and this could influence mycotoxin production on different commodities, as the microbial diversity of ripening crops will also change, which, in combination with more resilient toxigenic populations, could influence the amount and type of mycotoxins produced.

Recent studies of *F. verticillioides* and *F. graminearum*, which were cultivated for 10 generations under CC interacting abiotic conditions, were examined for growth and subsequent toxin production [78]. They found that for *F. verticillioides* and *F. graminearum*, the acclimatized strains had optimum growth at 30 °C + 0.98 a_w_ + 400 ppm CO_2_. Elevated temperature (35 °C and 33 °C respectively) and CO_2_ levels (800, 1200 ppm) did not significantly change fungal growth or mycotoxin production (i.e., FB_1_ and FB_2_ and ZEA). This indicated that these acclimatized strains were very resilient to exposure to the three-way interacting climate-related abiotic factors. More studies are necessary to better understand the ecophysiology of strains/species acclimatized to climate-related abiotic factors to improve our knowledge for prioritizing toxigenic fungi that represent an increased risk in the future.

## 7. Biocontrol Strategy, Resilience and Adaptation of a Fungal Antagonist to Climate Change—Prevention Methods in a New Risk Area

Biocontrol of fungal pathogens and pests of durable and horticultural crops, pre- and post-harvest, have received significant attention for many years. Biocontrol of mycotoxigenic pathogens are included in these research efforts. The mechanism of action has often been considered to include direct antagonism between the biological control agents (BCA) and fungal pathogen, competitive occupation by niche exclusion of the pathogen, or in combination with production of secondary metabolites, hyperparasitism, or with volatile organic compounds (VOCs). This approach has received significant impetus by the removal of a significant number of chemical groups for crop protection (50%) by the EU and the need for more sustainable systems in the context of the food security agenda. This has been a driver for the crop protection industry for the development of more integrated systems for disease and pest control including the use of BCAs. Development of BCAs for the control of mycotoxigenic fungi has been one of the focus areas [79,80]. However, the resilience of such control systems has not received significant attention [81,82,83]. Thus, for the development of effective BCAs, the resilience of biocompetitive strains under CC-related abiotic conditions has become critical for conservation of efficacy. Certainly, very little if any attention has been focused on screening and choosing strains that have the necessary resilience under future interacting abiotic conditions, e.g., elevated temperature (+2–5 °C), exposure to increased levels of CO_2_ (400 vs. 1000–1200 ppm CO_2_) or to extreme wet-dry episodes. Strains with the right resilience are required for efficacy under such interacting abiotic CC-related factors.

The timing of the application of BCAs for potential mycotoxin biocontrol was suggested to be critical in both groundnuts and maize, to ensure that the non-toxigenic strains competed effectively with the resident toxigenic ones. Recently, Kagot et al. [84] suggested that pre-harvest control of *Fusarium* mycotoxins (e.g., fumonisins) has not been very effective because of the lack of consistent field-based studies with species of *Trichoderma*, *Bacillus* and non-toxigenic *Fusarium* strains. However, many of these strains may not have the necessary resilience required for efficacy. In addition, previous studies with *Clonostachys rosea* showed that in milled maize media and maize grain, the water availability influenced the level of control achieved with different ratios of *F. verticillioides* and the BCA [85]. This showed that under drought stress, fumonisin B_1_ control was less effective because of the lack of resilience of the BCA. Subsequent studies with selected fungal and bacterial strains and using different ripening stages of maize cobs showed that there was good efficacy of both fungal and bacterial BCAs for FB_1_ control [85]. They showed that the ripening stage a_w_ of the kernels of the cob changed from R3, Milk (0.985 a_w_) to R4, Dough (0.976 a_w_) and R5, Dent (0.958 a_w_) stage. These are within the environmental a_w_ range for colonization by *F. verticillioides*. A BCA strain of *C. rosea* significantly reduced FB_1_ contamination of the maize cobs by >70% at 25 °C, and almost 60% at 30 °C regardless of maize ripening stage. For the bacterial antagonist, FB_1_ levels on maize cobs were significantly decreased, but only in some treatments, probably due to a lack of environmental resilience. In the case of bacteria, the requirement for relatively freely available water or a formulation that delivers enough water for replication and establishment is critical for efficacy to be maintained. Opportunities may thus exist for BCA identification and development for control of *Fusarium* mycotoxins, even in Low and Middle Income Countries (LMICs), provided effective and resilient strains can be identified and appropriate formulations developed.

Recent studies by Rodriguez-Sixtos [35] and Gasperini et al. [86] showed that when examining the effect of CC-related scenarios such as elevated CO_2_ and temperature (37 °C) in maize cobs of different ripening ages at R4 (0.976 a_w_) and R5 (0.958 a_w_) stages, using a 50:50 spore concentration of a non-toxigenic biocontrol *A. flavus* strain and a toxigenic strain, there was no significant AFB_1_ control. This again suggests that it is indeed critical to identify more resilient non-toxigenic strains of *A. flavus* for maintaining control of AFB_1_ production in staple cereals and groundnuts.

Once resilient strains have been identified then the production, and especially the formulation may become critical in conserving efficacy under CC-related scenarios. Under existing conditions, the major bottleneck in relation to relative tolerance to a wide range of water availabilities and temperatures and indeed tolerance of elevated CO_2_ needs to be borne in mind [81]. Indeed, the importance of ecological windows and resilience of candidate BCAs relative to the pathogen is critical [80,87,88]. However, few studies have screened candidate BCAs for resilience to such interacting abiotic conditions. Recent studies with different formulations of the BCA *Candida sake* showed that the three-way interacting CC-related abiotic factors influenced the viability of the yeast cells in different formulations. Thus, some formulations were more resilient than others under such abiotic stress factors [78,89]. Studies with other BCAs such as *Clonostachys rosea* and non-toxigenic strains of *A. flavus* for aflatoxin control in maize have also shown that biocontrol efficacy could be reduced or have no effect on the pathogen or toxin production [90]. Thus, it is critical that there is more focus on formulating BCAs, such that the resilience can be improved under the expected environmental changes that will affect both crop production and pest/disease control strategies.

## 8. Conclusions and Future Perspectives

Obtaining a clear picture of the influence of climate on overall crop contamination by mycotoxins and on toxigenic fungal ecology and adaptation is complex; thus the real impacts of CC will still be difficult to ascertain and will need more extended data and experiments both in vitro for the ecological aspects extended to multi-strain analyses, and in vivo with experimental trials associated to provisional models that need to be effectively validated by field data. In fact, a better understanding and modelling of the impact of CC scenarios requires additional experimental data. In addition, re-calibration of the models may well be necessary in relation to new information gained. Furthermore, geo-referenced field data are still particularly poor. There is also a need to try and merge/integrate the different models developed by different research groups for more robust assessment of relative toxin risks. It is very important to validate these models in different regions from those where the models were originally developed.

The advent of Next-Generation Sequencing (NGS) has greatly increased knowledge of the molecular aspects of the regulation of mycotoxin biosynthesis. However, for several toxigenic fungal species, data on toxin regulations at a molecular level under CC-scenarios are still very scarce. Genomic and transcriptomic approaches could contribute to fill these gaps, as well as the integration of epigenetic studies for understanding how chromatin remodeling is influenced under interacting environmental factors. This would provide more comprehensive insights into how CC may influence ecology and mycotoxin production by different toxigenic species.

Resilience under CC-related abiotic conditions and data on acclimatization to the predicted conditions are still scarce, so additional studies on toxigenic fungi acclimatization and interactions with other mycobiota are necessary to obtain more accurate data on implications for the predominance of mycotoxigenic fungi and mycotoxin contamination on staple food commodities under CC scenarios. In this respect, there are indications that changing climate conditions may lead to alterations in phenology and dynamics of fungal communities, but the impact of this global change on fungal ecology is still not very well understood. Acclimatization and resilience of biocontrol strains useful to counteract the mycotoxigenic fungi is indeed fundamental for the future of sustainable biological control of mycotoxin contamination in the field.

Increased temperatures and UV radiation may cause fungi to mutate on crops and produce different mycotoxins; studies on these aspects are difficult to carry out. However, this knowledge is needed to understand the relative tolerance of toxigenic species and whether the dominance of certain species complexes may occur under CC scenarios.

The availability of high quality data for statistical evaluation is an essential requirement, both for risk assessment studies as well as for studying the effect of CC on the prevalence of mycotoxins in food and feed chains at a global level. In particular, an accurate estimate of the impact of CC on mycotoxin incidence should be evaluated in the field, by analyzing raw materials (before any processing). Harmonized monitoring programs with harmonized sampling procedures and analytical method performances across the world over several growing seasons need to be conducted, possibly in the same field location and the same crop varieties. This will require multifactorial field experiment approaches under real-world conditions that consider the impacts of interactions of temperature, water and CO_2_ on toxigenic fungi in the field. In addition, it is highlighted that good agricultural and storage practices reduce fungal development as well as mycotoxin contamination. This aspect, as well as other mitigation strategies, should also be taken into consideration when data are analyzed and compared. Currently, there is relevant information on aflatoxins, deoxynivalenol, and ochratoxin A, and just a few studies have focused on others like fumonisins and *Alternaria* toxins. However, other mycotoxins need to also be considered and investigated under CC-scenarios. In addition, modified mycotoxins are commonly found in cereals and in processed foods, co-occurring with the native mycotoxins, as a result of fungal metabolism or the defense mechanism of the infected plant. This issue is of great interest due to the associated risks for human and animal health [91]. The effect of CC on the occurrence of modified mycotoxins is a neglected area and needs to be evaluated and included in future risk maps on a regional basis. By developing such research, it will be possible to have a better in depth understanding the impacts that climate-related abiotic factors have on food and feed production chains and develop appropriate intervention strategies to minimize the impact on feed safety and security.

Although during the last decade the international research community has been providing comprehensive and continuously updated knowledge and research on the potential effects that climate change may have on toxigenic fungi and mycotoxin risks, it is clear that there are important knowledge gaps that need further and deeper research studies. Multidisciplinary research is thus needed to address these new areas and to repeat and re-evaluate our current knowledge when we gain new insight into these biological systems.

## Figures and Tables

**Table 1:** Mycotoxins of major concern occurring in food/feed, relevant fungal producers and geographical areas affected.

Micotoxin	Matrix	Fungus	North America	Central-South America	Europe ^a^	Africa ^b^/Middle East	North Asia	South-East Asia
Aflatoxins (B_1_, B_2_, G_1_, G_2_)	maize, peanuts, nuts, spices, dried fruit	*A. flavus*, *A. parasiticus*	Occasional	Very frequent	Rare/Occasional	Very frequent	Rare	Frequent
Ochratoxin A	wheat, barley, maize, coffee, wine, beer	*A. ochraceus*, *A. carbonarius*, *A. niger*, *A. westerdijkiae*, *A. steynii*, *P. verrucosum*	Occasional	Rare	Frequent	Occasional	Occasional	Occasional
Deoxynivalenol	wheat, maize, barley	*F. graminearum* *F. culmorum*,	Frequent	Occasional	Frequent	Frequent	Frequent	Frequent
T-2/HT-2 toxins	wheat, maize, barley, rye, oats	*F. sporotrichioides*, *F. langsethiae*, *F. poae*	Occasional	Occasional	Frequent	Occasional	Frequent	Occasional
Zearalenone	maize, wheat	*F. graminearum*, *F. culmorum*, *F. crookwellense*	Occasional	Frequent	Frequent	Occasional	Frequent	Frequent
Fumonisins (B_1_, B_2_, B_3_)	maize	*F. verticillioides*, *F. proliferatum*	Very frequent	Frequent	Frequent	Very frequent	Very frequent	Very frequent

^a^ Northern Europe: AFs—rare, OTA—frequent, DON—frequent, T-2/HT-2—frequent, ZEA—occasional, FBs—frequent; Central Europe: AFs—rare, OTA—frequent, DON—frequent, T-2/HT-2—frequent, ZEA—frequent, FBs—frequent; Southern Europe: AFs—occasional, OTA—frequent, DON—frequent, T-2/HT-2—rare, ZEA—occasional, FBs—frequent; Eastern Europe: AFs—occasional, OTA—frequent, DON—frequent, T-2/HT-2—frequent, ZEA—frequent, FBs—frequent; ^b^ North Africa: AFs—occasional, OTA—frequent, DON—frequent, T-2/HT-2—rare, ZEA—occasional, FBs—very frequent; Sub-Saharan Africa: AFs—frequent, OTA—frequent, DON—frequent, T-2/HT-2—rare, ZEA—occasional, FBs—very frequent; South Africa: AFs—rare, OTA—rare, DON—frequent, T-2/HT-2—rare, ZEA—occasional, FBs—very frequent.
